# Unraveling changes in mitochondrial processes influenced by ancestry in *APOEε*4 carriers with Alzheimer disease

**DOI:** 10.3389/fnagi.2026.1888862

**Published:** 2026-09-28

**Authors:** M. D. M. Muniz Moreno, K. Celis, F. Rajabli, P. L. Whitehead, K. L. Hamilton-Nelson, D. M. Dykxhoorn, K. Nuytemans, M. M. Mesulam, D. A. Bennett, T. Schuck, S. Weintraub, C. Geula, M. Gearing, C. Alba, C. L. Dalgard, D. A. Davis, W. K. Scott, J. L. Haines, M. A. Pericak-Vance, A. J. Griswold, J. M. Vance, J. I. Young

**Affiliations:** 1 Hussman Institute for Human Genomics, University of Miami Miller School of Medicine, Miami, FL, United States; 2 Dr. John T Macdonald Foundation Department of Human Genetics, University of Miami Miller School of Medicine, Miami, FL, United States; 3 Northwestern ADC Neuropathology Core, Northwestern University Feinberg School of Medicine, Chicago, IL, United States; 4 Department of Neurological Sciences and Rush Alzheimer's Disease Center, Rush University Medical Center, Chicago, IL, United States; 5 The Department of Pathology and Laboratory Medicine, Institute on Aging and Center for Neurodegenerative Disease Research, The Perelman School of Medicine at the University of Pennsylvania, Philadelphia, PA, United States; 6 Goizueta Alzheimer’s Disease Research Center, Emory University, Atlanta, GA, United States; 7 Department of Anatomy Physiology and Genetics, Uniformed Services University, Bethesda, MD, United States; 8 Brain Endowment Bank, Department of Neurology, University of Miami Miller School of Medicine, Miami, FL, United States; 9 Cleveland Institute for Computational Biology, Case Western Reserve University, Cleveland, OH, United States

**Keywords:** Alzheimer disease, ancestry, APOE, mitochondria, single cell transcriptome

## Abstract

**Introduction:**

The *APOEε4* allele is the strongest known genetic risk factor for late-onset Alzheimer disease (AD). However, the risk effect of *APOEε4* varies substantially by ancestry, with individuals of African ancestry (AF) demonstrating markedly lower risk compared to those of European ancestry (EU). This difference in risk is associated with distinct levels of *APOE* gene expression. Here, we investigate whether mitochondrial processes in brain cells are also influenced by ancestry in AD *APOEε4* carriers, potentially contributing to the observed risk differences.

**Methods:**

Single nuclei RNA sequencing was performed on frontal cortex from 17 AD patients homozygous for *APOEe4* with either African Local Ancestry (AF-LA; *N* = 7) or European Local Ancestry (EU-LA; *N* = 10). Differential gene expression was compared between AF and EU samples, with particular focus on mitochondrial pathways. Differential expression and functional enrichment analyses were performed using MAST and PathFindR, respectively. MitoXplorer was used to identify differential mitochondrial processes, while REACTOME facilitated the building of regulatory networks.

**Results:**

Multiple cell types and biological processes contribute to the differences in *APOEe4* risk seen between individuals of AF and EU ancestry. AF astrocytes show a less reactive phenotype and L6 and L5/6 excitatory neurons appear to be less vulnerable in AF compared to EU samples. Mitochondrial alterations were more pronounced in EU-LA *APOEe4* AD carriers relative to their AF counterparts. Cells highly expressing *APOE* (astrocytes, microglia, and vascular leptomeningeal cells) showed increased expression of mitochondrial genes, particularly in EU-LA samples. We observed increased expression of pro-apoptotic, oxidative phosphorylation, and mitophagy-related genes in EU samples. Regulatory network analysis identified the transcription factor PPARG as a potential mediator linking *APOEε4* expression to mitochondrial gene programs in a cell-type- and ancestry-dependent manner.

**Conclusion:**

Our snRNA-seq analysis demonstrates that ancestry-linked regulation of *APOEε4* expression influences mitochondrial transcriptome and astrocyte activation in AD. The stronger *APOE–PPARG*–mitochondrial gene coupling in EU ancestry particularly in astrocytes may underlie increased AD risk, while AF ancestry confers partial decoupling of these pathways. These results underscore the importance of considering ancestry in AD research and suggest that precision therapeutic strategies targeting mitochondrial and glial pathways should account for *APOE* local ancestry.

## Background

Alzheimer’s disease (AD) is the most common neurodegenerative disorder and is characterized by amyloid-β (Aβ) plaques, neurofibrillary tau tangles, and widespread molecular and metabolic dysfunction, including impaired mitochondrial homeostasis (Iqbal and Grundke-Iqbal, 2010; Bell et al., 2021). AD affects approximately 6.7 million Americans over the age of 65 and, without effective disease-modifying therapies, is projected to affect more than 150 million individuals worldwide by 2050 (Alzheimer’s disease facts and Figures, 2024).

The *APOE* gene is the strongest genetic risk factor for late-onset AD. While the APOEε3 allele is considered largely neutral, the APOEε4 allele substantially increases AD risk (Frieden and Garai, 2012; Corder et al., 1993). Although *APOE* is a risk factor for AD in all populations, the effect size of the risk conferred by the e4 allele varies across different ancestries (Rajabli et al., 2018; Farrer et al., 1997; Hendrie et al., 2014; Sahota et al., 1997; Tang et al., 1996; Hohman et al., 2016), with individuals of African ancestry (AF) showing lower risk compared to those of European (EU) ancestry (Rajabli et al., 2018; Hendrie et al., 2014; Tang et al., 1996; Hohman et al., 2016). We and others have shown that this ancestry-specific difference in risk is linked to local ancestry (LA) surrounding the *APOE* gene (Rajabli et al., 2018; Blue et al., 2019). Further, we demonstrated that EU LA in *APOE*ε4 homozygous carriers resulted in elevated *APOE*ε4 expression compared to AF LA carriers in postmortem brain tissue (Griswold et al., 2021). This difference in *APOE*e4 expression was consistent with ancestry-specific differences in chromatin accessibility between these groups (Celis et al., 2023).

Beyond its established roles in lipid transport and amyloid biology, *APOE*ε4 has been strongly implicated in metabolic and mitochondrial pathways that contribute to neurodegeneration (Grootendorst et al., 2005; Liu et al., 2013; Husain et al., 2021; Chen et al., 2022; Tambini et al., 2016; Sienski et al., 2021; Cai and Tammineni, 2016). Studies have linked *APOE*ε4 to impaired mitophagy, altered calcium homeostasis, abnormal mitochondrial dynamics, increased oxidative stress, and bioenergetic deficits that emerge decades before clinical symptom onset (Cai and Tammineni, 2016; Mahley and Huang, 2012; Reiman et al., 1996; Williams et al., 2020). Mechanistic investigations further suggest that *APOE*ε4-derived fragments can directly interact with mitochondrial proteins, influence mitochondria-associated ER membranes, and regulate transcriptional programs involved in inflammation, aging, and cellular stress responses (Chang et al., 2005; Chen et al., 2011; Tambini et al., 2016; Theendakara et al., 2016; Schmukler et al., 2020). Growing evidence also supports a reciprocal relationship in which mitochondrial dysfunction can influence APOE expression and downstream pathogenic pathways (Schmukler et al., 2020).

Recent single-cell and spatial transcriptomic studies have demonstrated that APOE isoforms shape cell type-specific transcriptional responses in AD, particularly in astrocytes and microglia (Murphy et al., 2025). Reactive astrocyte populations increase with disease progression and exhibit distinct molecular states associated with neuroinflammation and metabolic dysfunction (Serrano-Pozo et al., 2024; Mathys et al., 2024; Lana et al., 2024). In parallel, mitochondrial-omics studies have identified disrupted bioenergetic pathways, oxidative stress, and impaired mitochondrial quality control as prominent features of the APOEε4 context (Perkins et al., 2016; Jiang et al., 2025). Emerging multiomic analyses further highlight ancestry-dependent molecular signatures, particularly within astrocytes, vascular cells, and microglia, emphasizing the importance of genetic background in shaping APOEε4 biology (Yilmaz et al., 2025; Mahley, 2023).

These converging lines of evidence raise a compelling question: Do ancestry-driven differences in *APOEε4* expression influence mitochondrial function and cell-type specific transcriptomic programs? To address this hypothesis, we analyzed single-nucleus transcriptomic data from autopsy brain samples derived from individuals homozygous for *APOE*ε4 with either AF or EU local ancestry. We focused on cell-type specific gene expression within mitochondrial pathways and examined *APOE* interactions with mitochondrial genes via protein–protein networks, regulatory network inference, and expression correlation analyses. We confirmed that EU ancestry samples showed higher *APOE* expression across several glial and vascular cell types, which was accompanied by stronger activation of mitochondrial gene programs. Network analyses highlighted PPARG as a shared regulator linking *APOE* and mitochondrial pathways, and trajectory analysis revealed that astrocytes from European samples progressed further toward a reactive state. Together, these results suggest that ancestry-linked regulation of *APOEε4* expression influences mitochondrial function and astrocyte activation in AD. Our findings underscore the importance of considering ancestry when investigating the molecular mechanisms of AD, particularly those involving *APOE* and mitochondrial biology.

## Materials and methods

### Brain samples

Seventeen brain samples were obtained as part of a multi-center collaboration from four Alzheimer Disease Research Centers (ADRCs; Northwestern University, Emory University, the University of Pennsylvania, and Rush University Medical Center) and from the John P. Hussman Institute for Human Genomics (HIHG) at the University of Miami Miller School of Medicine. All ADRC samples were selected using a previously described filtering criteria (Griswold et al., 2021). All samples were obtained in compliance with each institutional approved review board protocols at each institution. Eleven samples were already described in our previous reports (Griswold et al., 2021; Celis et al., 2023) (Table 1). Genotyping arrays were used to identify both local genetic ancestry (LA) in the *APOE* region and global genome-wide ancestry for each sample. The local ancestry of the *APOE* allele was defined as the region of 1 Mb on either side of *APOE* (chr19:44–46 Mb) as previously described (Rajabli et al., 2018). Global ancestry analyses were performed as described (Griswold et al., 2021). Whole genome sequencing (WGS) was performed on samples from individuals found to be homozygous for the EU or AF haplotype in the *APOE* LA region at the HIHG or The American Genome Center at Uniformed Services University of the Health Sciences (USUHS) using the TruSeq DNA PCR-Free library preparation kit (Illumina) with sequencing on the Novaseq 6000 (Illumina, San Diego, CA). The WGS output was processed using published best practices (McKenna et al., 2010) with alignment to the GRCh38 genome build with STAR, duplicate marking, base-quality recalibration and genotype calling with HaplotypeCaller.

**Table 1:** Clinical and genetic information of the patients included in the metadata.

Sample	Center	Sex	AOD	APOE genotype	Local Ancestry (Chr19: 44–46 Mb)	Global ancestry	BRAAK Score	Samples previously published?
1	Rush	Male	76	4,4	EU/EU	97% EU	IV	Celis et al., 2022
2	Rush	Female	86	4,4	EU/EU	98% EU	VI	Celis et al., 2022
3	Rush	Male	71	4,4	EU/EU	98% EU	VI	Celis et al., 2022
4	UM/Duke	Male	75	4,4	EU/EU	95% EU	IV	Celis et al., 2022
5	UM/Duke	Female	70	4,4	EU/EU	95% EU	VI	Celis et al., 2022
6	UM/Duke	Female	76	4,4	EU/EU	96% EU	V	Celis et al., 2022
7	Emory	Female	72	4,4	EU/EU	98% AF	VI	This study
8	Emory	Female	83	4,4	EU/EU	99% AF	VI	This study
9	Emory	Female	76	4,4	EU/EU	94% AF	VI	This study
10	Duke	Female	75	4,4	EU/EU	96% AF	IV	This study
11	Rush	Male	93	4,4	AF/AF	68% AF	V	Celis et al., 2022
12	Rush	Female	77	4,4	AF/AF	85% AF	VI	Celis et al., 2022
13	NW	Female	85	4,4	AF/AF	86% AF	V	Celis et al., 2022
14	Emory	Female	82	4,4	AF/AF	87% AF	V	Celis et al., 2022
15	Emory	Female	80	4,4	AF/AF	88% AF	VI	Celis et al., 2022
16	Rush	Male	83	4,4	AF/AF	78% AF	VI	This study
17	Emory	Male	86	4,4	AF/AF	94% AF	IV	This study

Key: AOD, Age of Death; UM, University of Miami; NW, Northwestern University; RUSH, RUSH University Medical Center; AF, African; EU, European.

### snRNA-seq sequencing

Nuclei were isolated from frozen frontal cortex brain tissue (Brodmann area 9) as previously described (Griswold et al., 2021; Celis et al., 2023). SnRNA-Seq was performed at the HIHG Center for Genome Technology as previously described (Griswold et al., 2021). Quality control steps are summarized in Supplementary methods.

### Seurat default steps for snRNA-Seq and cell type identification

Downstream analyses of scRNA-Seq were performed in the R programming environment using a Seurat-based pipeline (Hao et al., 2021) as described in the Supplementary methods. We set the optimal resolution parameter (res) to res = 0.5 and identified 42 distinct clusters based on the expression of well-known cell markers and the top 10 marker genes defining each cluster. This resolution allowed discrimination of four distinct astrocytes subpopulations: one reactive, two resting and one canonical as previously described (Habib et al., 2020; Sadick et al., 2022). Neurons, oligodendrocytes, microglia, endothelial and VLMC were well resolved based on published data (Luo et al., 2022), the Allen Brain Atlas (Shen et al., 2012), and Azimuth human motor cortex cell markers (Bakken et al., 2021).

### Differential expression and differential functional analyses

Differential expression analysis was performed using the MAST framework (Finak et al., 2015) with an adjusted *p*-value <0.05 and log_2_FC of the difference between conditions (AF vs. EU, females AF vs. females EU or males AF vs. males EU) higher in absolute value than 1. Those genes are noted as differentially expressed genes (DEGs). We chose the MAST method because it was developed for single cell analyses and allows to analyze complex experimental designs. We performed this analysis in all samples, i.e., AF vs. EU, only males AF vs. males EU and only females AF vs. females EU. The functional analysis of the DEGs per cell cluster was performed using PathFindR (Ulgen et al., 2019) querying REACTOME, KEGG and GO GeneSets, molecular function (MF), cellular compartment (CC), and biological processes (BP) databases. Then the pathways were grouped into 29 higher functional categories or meta-pathways to decrease the complexity of the analysis and get an overview of the functions more altered as described (Duchon et al., 2021).

### Mitochondrial pathways analysis

We downloaded the curated human interactome of genes involved in Mitochondrial function (noted as MT-genes) from MitoXplorer 2.0 database version 2022 (Marchiano et al., 2022). This version contains 1,229 genes annotated into one of 39 possible mitochondrial processes (noted MtFx). Following a gene set enrichment analysis approach (Luo et al., 2009) we considered as differentially expressed (DE) MT-genes those genes that demonstrated a statistically significantly change in expression between ancestries (adj *p*-value <0.05) and log2FC threshold (−0.32 < log_2_FC > 0.25). This allowed us to decipher if there are changes in the MtFx processes that could be due to small, coordinated changes in expression of several members of the processes instead of considering fewer strong deregulated genes. Although the presence of transcripts encoded in the mitochondrial genome is limited in snRNAseq data (cells with more than 20% mitochondrial transcripts were excluded from the analysis), we quantified the percentage of counts belonging to MT-genes and just mitochondrial-encoded genes using the Seurat PercentageFeatureSet function.

### Pairwise correlation analysis at the single cell level

We performed gene–gene expression correlation analyses without dropouts or imputation. This allows us information about the number of cells and number of independent measurements used to compute the final correlation score and significance *p*-value metrics and to omit r unmeasured expression as zero expression. Thus, we implemented a novel pipeline where first, per cluster, we selected cells that cells expressing at least one of the genes of interest. Second, for each pair of genes, we selected cells with non-zero expression of both genes and computed pairwise correlation using the rcorr function from the R package Hmisc.^1^ The final metrics provide the *p*-value, correlation score, the number of cells used for pairwise correlation, and the number of cells where non-zero expression was captured for the genes of interest.

### Network connectivity analysis

We built gene connectivity networks using the protein–protein interaction information stored in STRING database (Szklarczyk et al., 2017) and the regulatory information contained in REACTOME. We used NetworkAnalyst (Xia et al., 2015) to generate a preliminary minimum network based on the PPI selected from STRING DB by fixing a medium confidence threshold to select the PPI that were experimentally validated. The minimum network was exported to Cytoscape 3.5.1 (Shannon et al., 2003). The regulatory network was built using ReactomeFIViz app within the Cytoscape environment (Wu and Haw, 2017). Then, both the PPI and regulatory networks were merged to create the final protein–protein interaction and regulatory gene connectivity networks.

### Trajectory analyses using monocle3

We analyzed the dynamic changes in gene expression using monocle3 gene to produce a “trajectory” of cells’ differentiation (Trapnell et al., 2014). We analyzed the changes both in the number of cells per cluster that show a trajectory change and the overall rate of gene expression changes looking to ancestries differences within all the samples per ancestry. We performed this analysis on astrocytes because of their high expression of *APOE* and their relevant role in neurodegenerative diseases.

## Results

### Brain samples

In total, 17 AD brain samples were used for this study (Table 1), including 11 females (seven with AF and four with EU global ancestry) and six males (three with AF and three with EU global ancestry). The dorsolateral prefrontal cortex Brodmann area 9 (BA9), was used in this study since is a brain region consistently affected in AD and has been widely utilized in transcriptomic and single-cell studies of neurodegeneration. Its involvement in higher cognitive functions makes it particularly suitable for investigating ancestry-associated molecular differences in Alzheimer’s disease. All samples had BRAAK staging scores ranging from IV to VI. Mean age of death was 79 ± 3 years. Homozygosity for *APOEε4* and an absence of known variants in Mendelian genes for AD (*APP, MAPT, PSEN1*, and *PSEN2*), as well as absence of known rare AD risk variants in *ABCA7*, *SORL1*, and *TREM2* was confirmed by Sanger sequencing. All samples were homozygous for local ancestry (either EU or AF) surrounding *APOE*. Global ancestry analysis revealed that admixed ancestry blocks in the rest of the genome were randomly distributed. European samples had 95–98 percent European global ancestry, while African samples averaged 84% African ancestry (68% to 94%) and minor European ancestry blocks; all samples have matching global and local ancestry for the *APOE* locus.

### Ancestry specific change in abundance of astrocytes and neurons

We integrated 112,464 cells across the 17 samples and built a cell map containing 43 cell clusters (Figure 1A). These clusters include 13 clusters of excitatory neurons (Supplementary Figure 1), 8 clusters of inhibitory neurons (Supplementary Figure 2A), 12 clusters of oligodendrocytes (Supplementary Figure 2B), 4 clusters of astrocytes (c1 and c7 as resting astrocytes, c4 expressing canonical markers and c8 expressing markers of reactivity, Supplementary Figure 3), 2 microglia clusters (Supplementary Figure 2C), one cluster of oligodendrocyte progenitor cells (OPCs) (Supplementary Figure 2D), 2 clusters of Vascular Leptomeningeal Cells (VLMCs) (Supplementary Figure 2E), and one cluster of endothelial cells (Supplementary Figure 2F). The proportion per aggregated cell type is similar to other independent brain snRNA-Seq datasets (Supplementary Table 1, Kim et al., 2023; Huuki-Myers et al., 2024). No sample bias was observed in any of the clusters (Supplementary Figure 4).

**Figure 1:**
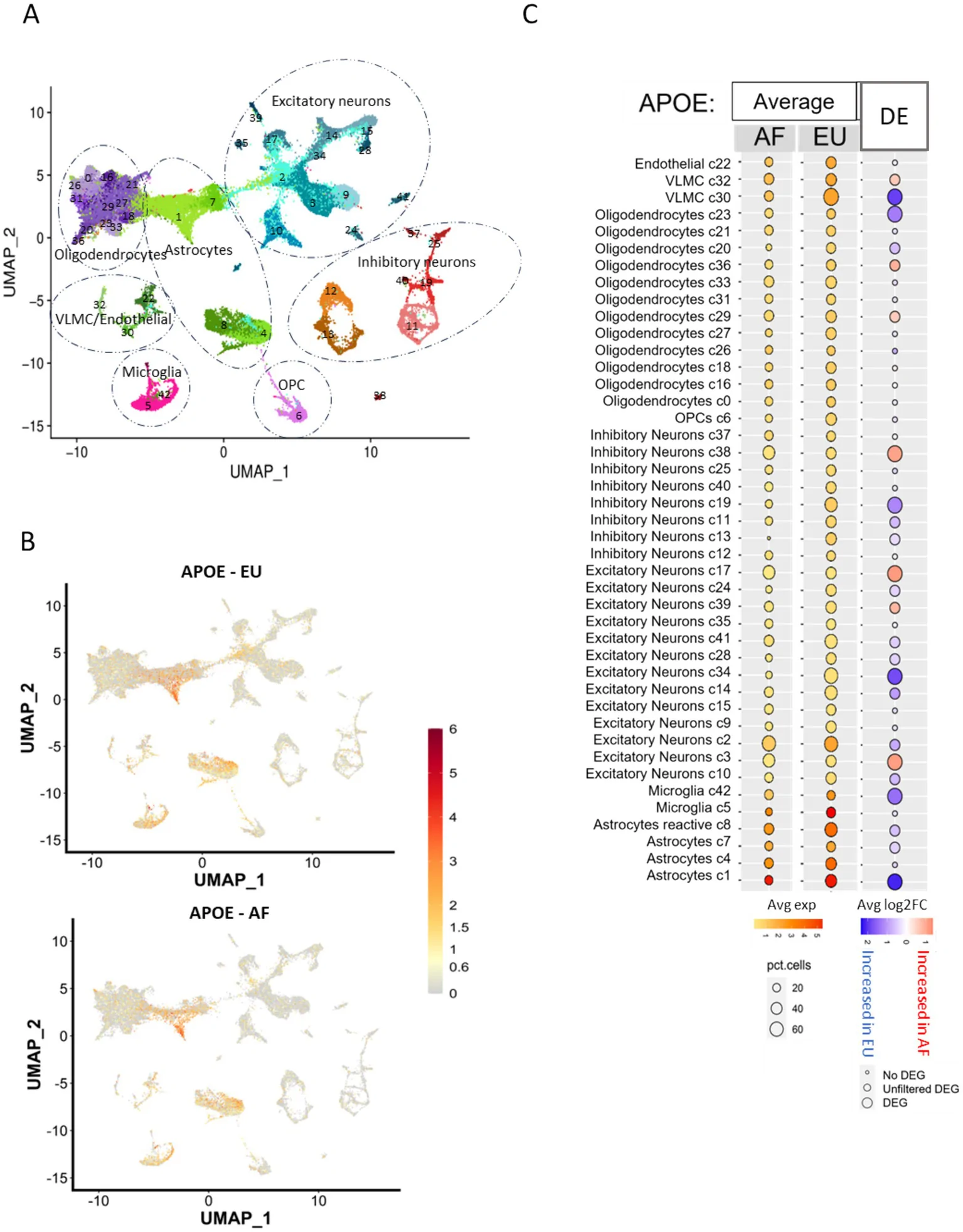
Cell clustering and *APOE* expression across single-nucleus transcriptomic profiles. **(A)** UMAP visualization of nuclei from 17 postmortem brain samples showing the 43 clusters identified using a clustering resolution of 0.4. Each color represents a distinct cell cluster. **(B)** UMAP visualization colored according to *APOE* expression levels across all nuclei. Top panel includes 10 EU and bottom panel 7 AF BA9 samples. The color scale indicates normalized gene expression. **(C)** Heatmap showing APOE expression levels across clusters stratified by ancestry (left two columns) and differential expression between EU and AF local ancestry groups (right column). Differential expression was assessed using MAST, with significance determined after multiple-testing correction (adjusted *p*-value <0.05). Colors indicate relative expression levels and direction of ancestry-associated differences.

Although most cell types exhibited similar proportion between ancestries, astrocyte cluster c7 had twice the number of cells in the EU samples while excitatory neuron clusters c39 and c41, identified as neurons from cortical layers 5 and 6 showed a 2-fold increase in the AF compared to EU samples (Supplementary Table 2, Supplementary Figure 5).

### Ancestry-related differential expression of APOE

*APOE* was expressed in the same cell clusters in both EU and AF samples (Figure 1B) and showed differential expression between EU and AF LA samples (Figure 1C, Supplementary Table 3). *APOE* showed a significant change in expression in 9 clusters with an absolute fold change >1. Specifically, *APOE* expression was elevated in the EU samples from astrocyte cluster c1, microglia cluster c42, oligodendrocyte cluster c23, and VLMC cluster c30, as previously described (Figure 1C) (Griswold et al., 2021). Higher expression of *APOE* in EU samples was also seen in neuronal clusters c34 and c19, while neuronal clusters c3, c17, and c38 showed elevated *APOE* expression in the AF samples.

### Ancestry, APOE expression and mitochondrial gene expression

We observed that clusters with the highest expression of *APOE* also showed the highest expression of mitochondrial genes (MT-genes) – encompassing both MT-encoded and nuclear encoded genes with mitochondrial function – compared to non-MT genes (Kruskal–Wallis chi-squared = 13,393, *p*-value < 2.2E-16; Figure 2). Although the elevated level of MT-genes in these high *APOE* expressing clusters was consistent across both ancestries (Figure 2B, Kruskal–Wallis with Dunn’s multiple comparison test: AF high expressing *APOE* cells vs. low *APOE* cells Z-statistics = 68, adj. *p*-value <1E-50; EU high *APOE* cells vs. low expressing *APOE* cells Z-statistics = 91, adj. *p*-value<1E-50), cells from EU individuals showed a higher relative increase in MT-gene expression compared to those from AF samples (Dunn’s test: AF vs. EU high expressing *APOE* cells Z-statistic = −11, adj. *p*-value = 1.57 E-32; AF vs. EU low expressing *APOE* cells Z-statistic = −14, adj. *p*-value = 4.08 E-47).

**Figure 2:**
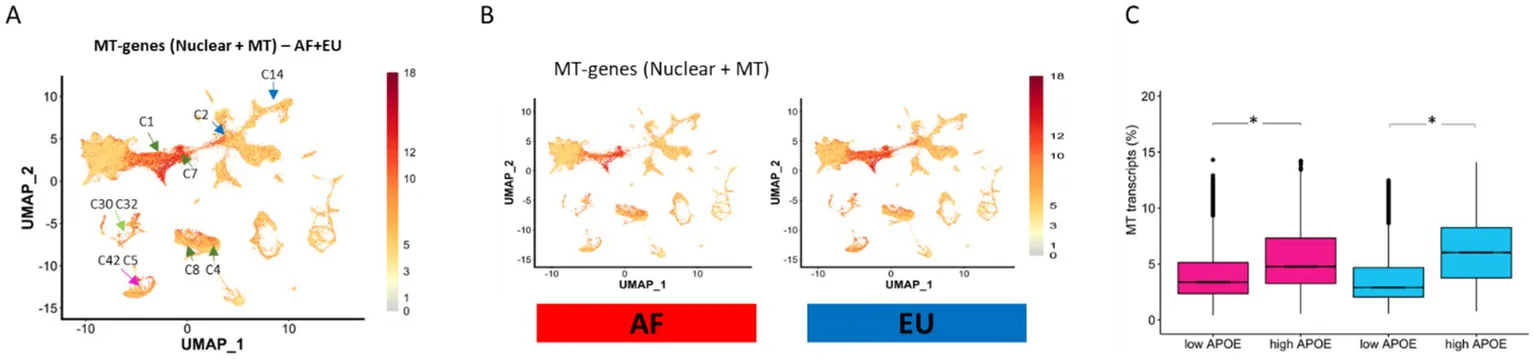
Mitochondrial gene expression patterns across ancestry groups and APOE expression levels. **(A)** UMAP visualization of the integrated single-nucleus RNA-seq dataset derived from 17 postmortem brain samples, colored according to aggregated mitochondrial gene expression scores, including both nuclear-encoded and mitochondrial genome-encoded mitochondrial genes. The color scale indicates relative expression levels. **(B)** UMAP visualization of nuclei from AF (*n* = 7, left) and EU (*n* = 10, right) local ancestry groups, colored according to aggregated mitochondrial gene expression scores. The color scale represents normalized mitochondrial gene expression. **(C)** Boxplots showing the percentage of mitochondrial transcripts in nuclei with high versus low *APOE* expression stratified by ancestry. Boxes indicate the interquartile range, center lines represent the median, and whiskers denote 1.5 × the interquartile range. Statistical significance was determined using a two-sided Wilcoxon rank-sum test. Test, and adj *p*-value <0.05 are shown on the plot as asterisks.

We then tested whether MT-gene expression differed globally between ancestries and detected significant differential expression across the majority of clusters. Altogether, we observed differential expression of 184 MT-genes between ancestries (adj *p*-value <0.05, −1 < log2FC > 1, Supplementary Table 4). Some of these differentially expressed (DE) genes were found broadly across multiple clusters (as many as 27 clusters) while other DE genes were unique to a single cluster. The MT-genes found to be DE in multiple clusters show a strong correlation in the direction of change across the different cell types (Supplementary Figure 6), suggesting a highly synchronized transcriptional program among these DE genes across the distinct cell clusters.

Using MitoXplorer, we found that transcriptional changes between ancestries were significantly enriched for genes involved in critical mitochondrial functions (Supplementary Table 5). These pathways include signaling (Calcium Signaling & Transport, enriched in 24 clusters), quality control (Mitophagy, 13 clusters) and bioenergetics (Pyruvate Metabolism, 16 clusters and Fatty Acid Degradation & Beta-oxidation, 10 clusters) processes.

### Regulation of gene expression in clusters with high correlation of APOE with MT-genes

To investigate the relationship between *APOE* expression, mitochondrial (MT) genes, and mitochondrial processes, we constructed regulatory networks for clusters exhibiting both ancestry-associated differences in *APOE* expression and overall high *APOE* expression levels (astrocyte cluster c1, microglia cluster c42, and VLMC cluster c30). For each of these clusters, we included *APOE* and MT-genes that were differentially expressed between ancestries (adjusted *p* < 0.05; |log₂FC| > 0.32; Supplementary Table 4) in the network building.

Across all three networks, *APOE* expression was directly linked to the differentially expressed transcription factor *PPARG* (Figure 3). Consistent with this connection, single-cell expression analyses revealed a strong correlation between *APOE* and *PPARG* (Supplementary Table 6) in European-ancestry astrocyte (c1, r^2^ = 0.52, *p* = 0.004) and microglia (c5, r^2^ = 0.17, *p* = 0.017) cells, as well as in African-ancestry microglia (c5, r^2^ = 0.45, *p* = 0.003), but not in African-ancestry astrocytes (c1, r^2^ = 0.25, *p* = 0.27). Through this first-degree interaction, *APOE* may influence approximately 8%–11% of the MT-gene network, depending on the cell type.

**Figure 3:**
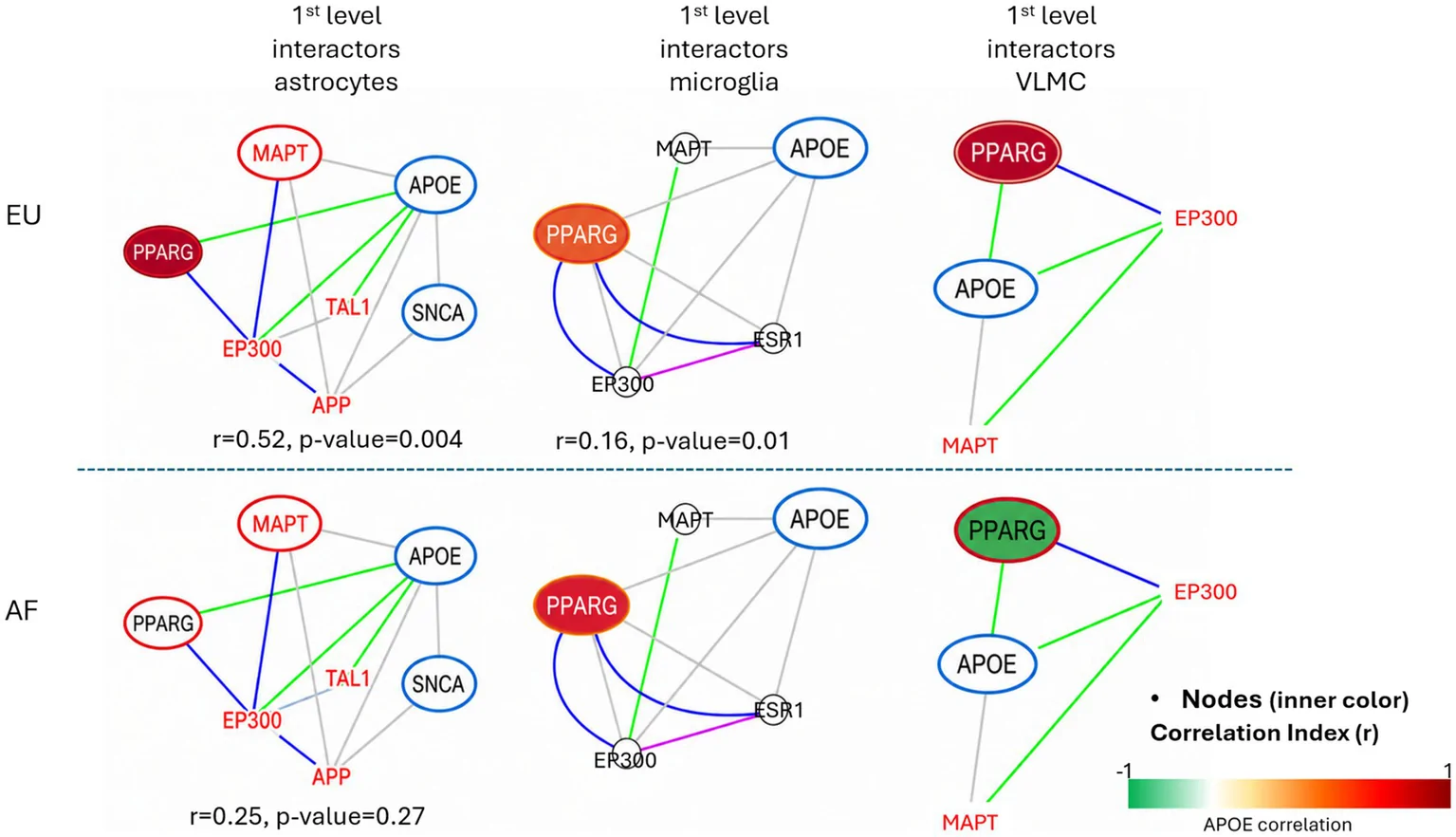
Mitochondria-related functional regulatory networks associated with APOE across cell types. Mitochondrial Functional Regulatory Networks (MtFxRegNet) constructed for astrocytes (left), microglia (middle), and vascular leptomeningeal cells (VLMCs; right). The top row shows networks derived from individuals with EU local ancestry, whereas the bottom row shows networks from individuals with AF local ancestry. Networks display the first-degree interaction partners of *APOE* within mitochondria-associated regulatory pathways. Node color represents the mitochondrial functional score, with warmer colors indicating higher functional relevance and cooler colors indicating lower functional relevance.

### Ancestry-specificity in trajectory from resting to reactive astrocytes

Since we found increased and correlated expression of *APOE*, *PPARG*, and MT-genes in astrocytes from EU brain samples and it has been previously reported that these factors modulate astrocyte activation, (Arnaud et al., 2022; Bernardo and Minghetti, 2008; Xiong et al., 2022) we performed trajectory analysis using monocle3 to see if there were ancestry-specific differences along the path from resting to reactive astrocytes (Figure 4A). We identified Astrocytes c1 (resembling resting astrocytes) as the starting cell cluster and the reactive subpopulation c8 as the end cluster in both ancestries. In support of the trajectory progression defined by monocle3, the differential gene expression analysis shows c1 DEG profile more closely resembles the c4 cluster than that of c8 (Figure 4B, Supplementary Table 3). When comparing the trajectory between ancestries, we observed a decrease in pseudo-time in the AF compared to EU samples (pseudo-time AF = 1.6, pseudo-time EU = 2, Figure 4C). The trajectory to reactive astrocytes shows a stronger divergence in gene expression profile in EU than in AF samples independent of the number of cells.

**Figure 4:**
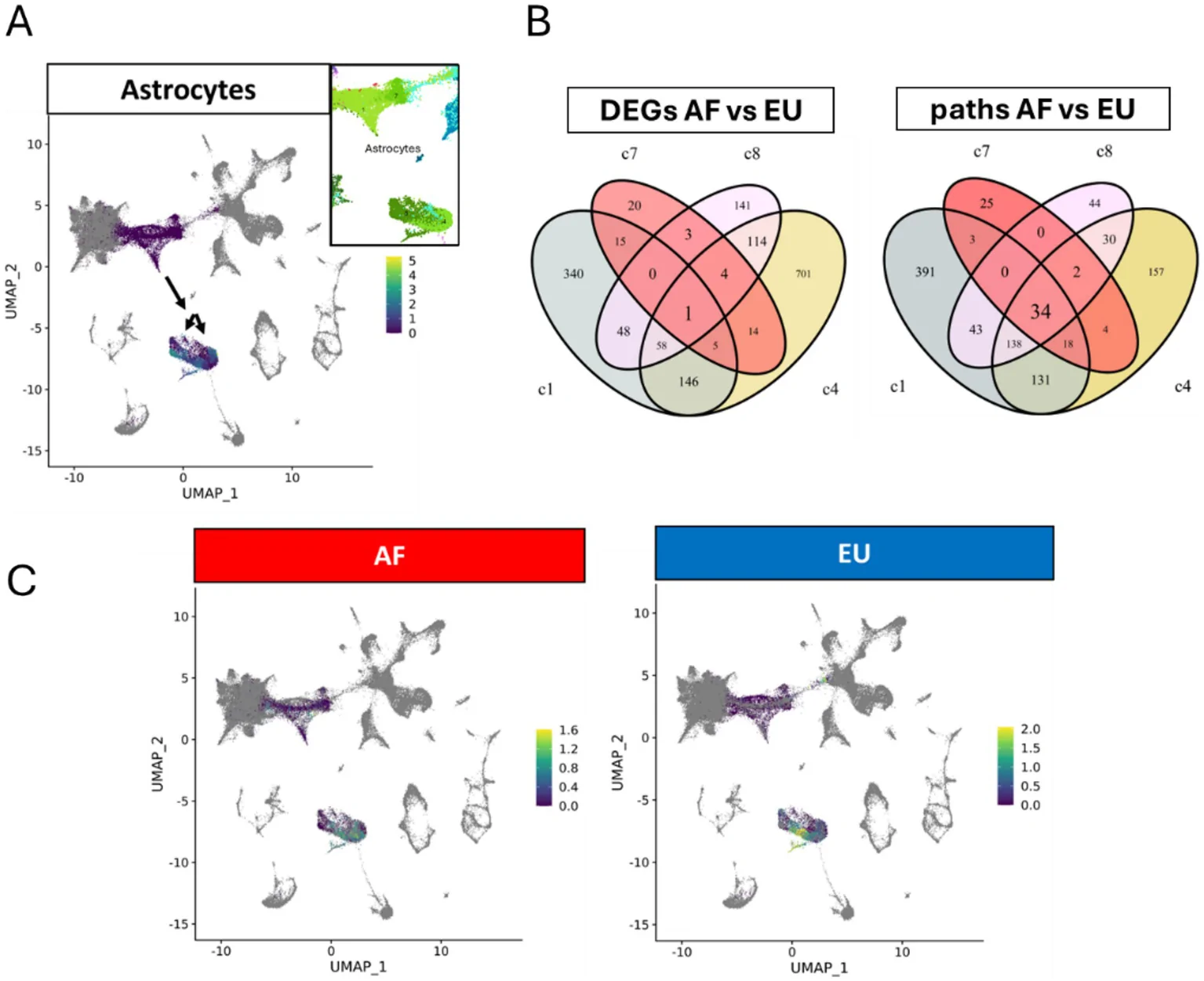
Astrocyte trajectory analysis reveals ancestry-associated differences in progression. **(A)** Pseudotime trajectory analysis of astrocyte subpopulations derived from 17 postmortem AD brain samples. Resting astrocyte clusters (c1, c4, and c7) are connected along a trajectory leading toward the reactive astrocyte cluster (c8). Nuclei are colored according to pseudotime progression, with darker colors indicating earlier states and lighter colors indicating later states. The inset shows the corresponding astrocyte cluster identities on the UMAP projection. **(B)** Venn diagrams showing the overlap of differentially expressed genes (DEGs; left) and significantly enriched functional pathways (right) among astrocyte clusters c1, c4, c7, and c8 in comparisons between AF and EU local ancestry groups. Numbers indicate the count of unique and shared DEGs or pathways. Differential expression and pathway enrichment analyses were performed as described in the Methods section, with significance determined after multiple-testing correction. **(C)** Pseudotime trajectories stratified by local ancestry. Astrocytes from individuals with AF local ancestry (*n* = 7) are shown on the left and those with EU local ancestry (*n* = 10) on the right. Cells are colored according to pseudotime values, illustrating ancestry-associated differences in progression toward the reactive astrocyte state.

## Discussion

Leveraging single-nuclei transcriptomes from 17 late-stage AD brains matched for *APOE* genotype (ε4 homozygous) and local ancestry, we identified ancestry-related differences in cell-type composition, *APOE* expression, and mitochondrial gene programs that converge on a PPARG-centered regulatory axis. Together, these findings provide a mechanistic framework that helps explain, at least in part, the ancestry-specific differences in *APOE* risk we have identified and linked to elevated glial *APOEε4* expression in EU samples.

The recent study by Fortea and colleagues posited that *APOEε*4 homozygosity represents a distinct genetic form of AD with nearly complete penetrance and predictable biomarker progression (Fortea et al., 2024). However, this conceptualization was based primarily on individuals of European ancestry, and multiple analyses have confirmed that *APOEε*4’s effect size varies dramatically across populations (Rajabli et al., 2018; Farrer et al., 1997; Hendrie et al., 2014; Sahota et al., 1997; Tang et al., 1996; Hohman et al., 2016; Blue et al., 2019). Our work directly addresses this critical knowledge gap by examining the molecular mechanisms underlying ancestry-specific *APOEε*4 risk differences.

Although the data showed that major brain cell classes were represented without sample bias, we observed ancestry-specific differences in the abundance of specific cell populations. Notably, there was a 2-fold enrichment of astrocyte c7 in European samples and a 2-fold enrichment of deep cortical layer 5/6 excitatory neurons (c39 and c40) in African samples. Such compositional shifts are likely relevant because *APOE* expression and downstream pathways are highly cell-state dependent. Interestingly, neurons from deep cortical layers 5/6 have been recently identified as selectively vulnerable in AD models (Kazmi and Wierenga, 2025). Our data suggest that in AD individuals with AF LA, these cells are preferentially preserved, raising the possibility that *APOE* expression levels may impact survival of these cells, particularly under stress conditions which have been shown to drive an increase in *APOE* expression in neurons (Boschert et al., 1999). The increase in the number of astrocytes in cluster c7 in EU brains, likely representing resting astrocytes, could also contribute to *APOE* risk modulation. It has been shown that the transition of astrocytes from a resting state to a reactive state is a primary pathogenic event in the development of AD (Kumar et al., 2023). Our observation of divergent trajectories from resting to reactive astrocytes in EU-LA versus AF-LA samples (Figure 4) highlights an additional cellular mechanism potentially contributing to the differences seen in *APOEε4* AD risk between ancestries. Green and colleagues (Green et al., 2024) built a comprehensive cell atlas of the aged prefrontal cortex, identifying a cellular trajectory leading to AD that is distinct from other aging-related effects; causal modeling prioritized specific astrocyte subpopulations as mediators of the effect of tau on cognitive decline. Our ancestry-stratified trajectory results directly extend this framework: the broader activation landscape and greater pseudotime progression observed in EU-LA astrocytes suggests that ancestry-linked regulation of *APOE* expression may influence which trajectory a given individual follows. AF-LA astrocytes, with their more compact pseudotime and partial decoupling of lipid and mitochondrial programs (Joshi et al., 2025; Qi et al., 2021), may resist progressing fully down the AD-dementia-associated cellular trajectory.

A notable finding is the identification of a significant correlation between high expression of *APOEε4* with elevated expression of MT-genes at the cell cluster level. Overall, clusters with the highest levels of *APOE* expression exhibited the highest levels of MT-gene expression. Whether this a general phenomenon or seen only in AD *APOEε4* brains is unknown. Recent work has demonstrated that mitochondrial dysfunction itself can drive increased *APOE* expression through disruption of the electron transport chain, suggesting a bidirectional relationship (Gokhale et al., 2023). Recent experimental evidence has now directly demonstrated that *APOEε*4 disrupts mitochondrial bioenergetics in astrocytes by impairing respiration and over-activating glycolysis, with *APOEε*4 astrocytes also showing decreased mitochondrial membrane potential and an inability to utilize endogenous fatty acids for energy metabolism (Qi et al., 2021; Lysaker et al., 2025). This is consistent with our observation of EU-ancestry–elevated MT-gene expression in glial clusters and reinforces the idea that the cellular mitochondrial landscape in AD depends on the interaction between *APOE* genotype and cell state. Our differential gene expression analysis revealed ancestry-biased alterations in mitochondrial pathways relevant to AD, including apoptosis regulation, mitochondrial dynamics, ROS defense and oxidative phosphorylation. Since several cell clusters with significant differential expression of MT-genes did not show significant *APOE* expression differences, both ancestry and *APOE* could potentially be contributing to the observed differential mitochondrial expression levels. Whether these processes contribute to AD risk by affecting cellular vulnerability remains to be elucidated. However, it is tempting to hypothesize that ancestry-related elevated expression of *APOEε4* leads to altered mitochondrial function which in turn affects cellular vulnerability and, consequently, AD risk.

The coordinated directional expression seen across genes with DE in multiple clusters highlights a robust, multi-gene metabolic and transport program that is co-regulated in an ancestry-dependent manner across specific cell states. The regulatory network analysis implicated an interaction between *APOE* and the transcription factor *PPARG* as a key component to modulate the MT transcriptional landscape. PPARG (Peroxisome proliferator-activated receptor-γ) is a nuclear receptor that regulates numerous cellular functions, including adipogenesis, lipid biosynthesis, energy expenditure and storage, inflammation, and differentiation (Hernandez-Quiles et al., 2021). PPARG regulates transcription by binding to peroxisome proliferator response elements present in regulatory regions of target genes (Li et al., 2022). A number of metabolic functions are controlled by PPARG in association with the co-factor PPARGC1A, a master regulator of mitochondrial biogenesis (LeBleu et al., 2014). Notably, PPARγ knockdown in cell-based models results in upregulation of several late-onset AD-associated genes, with the greatest effect on *APOE* mRNA, and additional upregulation of *ABCA7*, *CASS4*, *CELF1*, *PTK2B*, and *ZCWPW1*, proposing PPARγ as a master regulator of the transcription of multiple genes involved in AD pathogenesis (Barrera et al., 2018). PPARG also serves as an important regulator of anti-inflammatory activity, acting in part by antagonizing the nuclear factor-κB (NF-κB) pathway. Treatment with the PPARG agonist pioglitazone induced *APOE* expression and promoted Ab clearance in both microglia and astrocytes in APPswe/PSEN1dE9 (APP/PS1) mouse model of AD (Mandrekar-Colucci et al., 2012). Further, Skerrett et al. (2015) showed that pioglitazone treatment resulted in decreased soluble and aggregated Aβ in the hippocampi, increased *APOE* expression, elevated anti-inflammatory effects, and improved behavioral outcomes (Skerrett et al., 2015). These studies and others support a role for PPARg in mediating key processes underlying AD pathogenesis, as well as contributing to *APOE* expression regulation. However, PPARG agonists (e.g., thiazolidinediones) have shown mixed results in preclinical and clinical AD studies. Our data suggest a potential explanation, the efficacy may depend on cell type and *APOE* local ancestry and underlying mitochondrial/immune programs they influence. In EU-LA contexts, where *APOE*–*PPARG*–MT coupling is stronger, astrocyte-targeted PPARG modulation might yield larger effects, whereas in AF-LA contexts responsiveness may be attenuated. Stratifying trials by *APOE local* ancestry and enriching for astroglial delivery could clarify this signal.

Literature supports that *APOEε4* biases astrocytes toward pro-inflammatory, metabolically costly states and that astrocyte metabolism (lipid flux, β-oxidation, glutamate–lactate shuttling) shapes reactive heterogeneity. Our trajectory results, together with stronger *APOE*–*PPARG*–MT coupling in EU astrocytes, fit a model where EU-LA at *APOE* broadens the activation landscape—more mitochondrial remodeling and oxidative stress—as astrocytes react. AF astrocytes show a more compact trajectory, consistent with partial decoupling of lipid and MT programs.

One important limitation of our study is that our conclusions are based on transcriptional profiles and that future studies incorporating functional mitochondrial assays, such as measurements of oxidative phosphorylation, ATP production, reactive oxygen species generation, and mitochondrial membrane potential, will be required to establish the functional consequences of these ancestry-associated expression differences. We also acknowledge that although the cohorts were carefully selected and matched for residual differences related to age, sex, tissue processing procedures, postmortem interval, RNA integrity, and sample source may contribute to some of the observed transcriptional differences. This inherent limitation of postmortem human studies should be considered when interpreting ancestry-associated transcriptional signatures.

## Conclusion

Our data highlights a potential role for mitochondria-associated transcriptional signatures and cellular vulnerability in the risk conferred by *APOEε4* in different ancestral backgrounds. Our studies stress the importance of performing ancestry aware analyses at the single cell level in AD to fully capture genotype × ancestry × cell-state interactions, and to inform precision therapeutic and preventive strategies.

## Data Availability

The datasets presented in this study can be found in online repositories. The names of the repository/repositories and accession number(s) can be found in the article/Supplementary material.

## Footnotes

1
https://github.com/harrelfe/Hmisc

## Supplementary material

SUPPLEMENTARY FIGURE 1 Identification of excitatory neuron subpopulations. Comparative expression of excitatory neuron markers was utilized to classify 13 excitatory neuron clusters (see Supplementary Table 2). UMAPs colored based on selected neuronal markers.

SUPPLEMENTARY FIGURE 2 Identification of defined cell type subpopulations. Comparative expression of **(A)** inhibitory neuron, **(B)** oligodendrocyte, **(C)** microglia, **(D)** OPC, **(E)** VLMV and **(F)** endothelial cell markers was utilized to classify clusters (see Supplementary Table 2). UMAPs colored based on selected cell-type markers.

SUPPLEMENTARY FIGURE 3 Identification of astrocyte subpopulations. Comparative expression of astrocytic markers was utilized to classify 4 astrocyte clusters (see Supplementary Table 2). UMAP colored based on astrocyte markers.

SUPPLEMENTARY FIGURE 4 QC of the samples to assure no sample bias. UMAP representation of clusters colored per cell type. Left, AF samples. Right, EU samples.

SUPPLEMENTARY FIGURE 5 Proportion of cells per cluster per ancestry. Bars depict the percentage of cells belonging to a specific cluster with respect to all cells (In red AF cell proportions. In blue EU cell proportions). Arrows indicate clusters with different proportions between the ancestries.

SUPPLEMENTARY FIGURE 6 Coordinated directional expression of ancestry-biased differentially expressed genes across multiple clusters. Within any individual cluster, the selected genes show a highly unified direction of fold-change. For example, in cluster 4, both SLC44A1 and SLC25A13 exhibit elevated expression in African (AF) local ancestry relative to European (EU) samples. Conversely, in cluster 5, both transcripts show a synchronized inversion, displaying higher relative expression in EU individuals. **(A)** MT genes with higher expression in EU samples in cluster 7. **(B)** MT-genes with lower expression in EU samples in cluster 7.

## References

[ref1] Alzheimer’s disease facts and Figures (2024). 2024 Alzheimer's disease facts and figures. Alzheimers Dement. 20, 3708–3821. doi: 10.1002/alz.13809, 38689398 PMC11095490

[ref2] Arnaud L. Benech P. Greetham L. Stephan D. Jimenez A. Jullien N. . (2022). APOE4 drives inflammation in human astrocytes via TAGLN3 repression and NF-κB activation. Cell Rep. 40:111200. doi: 10.1016/j.celrep.2022.111200, 35977506

[ref3] Bakken T. E. Jorstad N. L. Hu Q. Lake B. B. Tian W. Kalmbach B. E. . (2021). Comparative cellular analysis of motor cortex in human, marmoset and mouse. Nature 598, 111–119. doi: 10.1038/s41586-021-03465-8, 34616062 PMC8494640

[ref4] Barrera J. Subramanian S. Chiba-Falek O. (2018). Probing the role of PPARγ in the regulation of late-onset Alzheimer’s disease-associated genes. PLoS One 13:e0196943. doi: 10.1371/journal.pone.0196943, 29723294 PMC5933777

[ref5] Bell S. M. Barnes K. De Marco M. Shaw P. J. Ferraiuolo L. Blackburn D. J. . (2021). Mitochondrial dysfunction in Alzheimer's disease: a biomarker of the future? Biomedicine 9:63. doi: 10.3390/biomedicines9010063, 33440662 PMC7827030

[ref6] Bernardo A. Minghetti L. (2008). Regulation of glial cell functions by PPAR-gamma natural and synthetic agonists. PPAR Res. 2008:864140. doi: 10.1155/2008/864140 PMC2367430 18464925

[ref7] Blue E. E. Horimoto A. R. V. R. Mukherjee S. Wijsman E. M. Thornton T. A. (2019). Local ancestry at APOE modifies Alzheimer's disease risk in Caribbean Hispanics. Alzheimers Dement. 15, 1524–1532. doi: 10.1016/j.jalz.2019.07.016, 31606368 PMC6925639

[ref8] Boschert U. Merlo-Pich E. Higgins G. Roses A. D. Catsicas S. (1999). Apolipoprotein E expression by neurons surviving excitotoxic stress. Neurobiol. Dis. 6, 508–514. doi: 10.1006/nbdi.1999.0251, 10600406

[ref9] Cai Q. Tammineni P. (2016). Alterations in mitochondrial quality control in Alzheimer's disease. Front. Cell. Neurosci. 10:24. doi: 10.3389/fncel.2016.00024, 26903809 PMC4746252

[ref10] Celis K. Moreno M. D. M. M. Rajabli F. Whitehead P. Hamilton-Nelson K. Dykxhoorn D. M. . (2023). Ancestry-related differences in chromatin accessibility and gene expression of APOE ε4 are associated with Alzheimer's disease risk. Alzheimers Dement. 19, 3902–3915. doi: 10.1002/alz.13075, 37037656 PMC10529851

[ref11] Chang S. Ma T. R. Miranda R. D. Balestra M. E. Mahley R. W. Huang Y. . (2005). Lipid-binding domain of apolipoprotein E4 enhances toroid-like structure formation and neurotoxicity of central nervous system neurons. Proc. Natl. Acad. Sci. USA 102, 18658–18663. doi: 10.1073/pnas.0508254102

[ref12] Chen H. Chen F. Jiang Y. Zhang L. Hu G. Sun F. . (2022). A review of APOE4 interference targeting Mitophagy molecular pathways for Alzheimer's disease. Front. Aging Neurosci. 14:881239. doi: 10.3389/fnagi.2022.881239, 35669462 PMC9166238

[ref13] Chen H. K. Ji Z. S. Dodson S. E. Miranda R. D. Rosenblum C. I. Reynolds I. J. . (2011). Apolipoprotein E4 domain interaction mediates detrimental effects on mitochondria and is a potential therapeutic target for Alzheimer disease. J. Biol. Chem. 286, 5215–5221. doi: 10.1074/jbc.M110.151084, 21118811 PMC3037634

[ref14] Corder E. H. Saunders A. M. Strittmatter W. J. Schmechel D. E. Gaskell P. C. Small G. W. . (1993). Gene dose of apolipoprotein E type 4 allele and the risk of Alzheimer's disease in late onset families. Science 261, 921–923. doi: 10.1126/science.8346443, 8346443

[ref15] Duchon A. Del Mar Muniz Moreno M. Martin Lorenzo S. Silva de Souza M. P. Chevalier C. Nalesso V. . (2021). Multi-influential genetic interactions alter behaviour and cognition through six main biological cascades in down syndrome mouse models. Hum. Mol. Genet. 30, 771–788. doi: 10.1093/hmg/ddab012, 33693642 PMC8161522

[ref16] Farrer L. A. Cupples L. A. Haines J. L. Hyman B. Kukull W. A. Mayeux R. . (1997). Effects of age, sex, and ethnicity on the association between apolipoprotein E genotype and Alzheimer disease. A meta-analysis. APOE and Alzheimer disease Meta analysis consortium. JAMA 278, 1349–1356. 9343467

[ref17] Finak G. McDavid A. Yajima M. Deng J. Gersuk V. Shalek A. K. . (2015). MAST: a flexible statistical framework for assessing transcriptional changes and characterizing heterogeneity in single-cell RNA sequencing data. Genome Biol. 16:278. doi: 10.1186/s13059-015-0844-5, 26653891 PMC4676162

[ref18] Fortea J. Pegueroles J. Alcolea D. Belbin O. Dols-Icardo O. Vaqué-Alcázar L. . (2024). APOE4 homozygosity represents a distinct genetic form of Alzheimer's disease. Nat. Med. 30, 1284–1291. doi: 10.1038/s41591-024-02931-w, 38710950 PMC13310155

[ref19] Frieden C. Garai K. (2012). Structural differences between APOE3 and APOE4 may be useful in developing therapeutic agents for Alzheimer's disease. Proc. Natl. Acad. Sci. USA 109, 8913–8918. doi: 10.1073/pnas.1207022109, 22615372 PMC3384159

[ref20] Gokhale A. Hartwig C. Freeman A. A. H. Bassell J. L. Zlatic S. A. Sapp Savas C. . (2023). Systems analysis of the 22q11.2 microdeletion syndrome converges on a mitochondrial interactome necessary for synapse function and behavior. eLife 12:e85779. doi: 10.1523/JNEUROSCI.1983-18.2019 PMC6495129 30833507

[ref21] Green G. S. Fujita M. Yang H. S. Taga M. Cain A. McCabe C. . (2024). Cellular communities reveal trajectories of brain ageing and Alzheimer's disease. Nature 633, 634–645. doi: 10.1038/s41586-024-07871-6, 39198642 PMC11877878

[ref22] Griswold A. J. Celis K. Bussies P. L. Rajabli F. Whitehead P. L. Hamilton-Nelson K. L. . (2021). Increased APOE ε4 expression is associated with the difference in Alzheimer's disease risk from diverse ancestral backgrounds. Alzheimers Dement. 17, 1179–1188. doi: 10.1002/alz.12287, 33522086 PMC8843031

[ref23] Grootendorst J. Bour A. Vogel E. Kelche C. Sullivan P. M. Dodart J. C. . (2005). Human APOE targeted replacement mouse lines: h-APOE4 and h-APOE3 mice differ on spatial memory performance and avoidance behavior. Behav. Brain Res. 159, 1–14. doi: 10.1016/j.bbr.2004.09.019, 15794991

[ref24] Habib N. McCabe C. Medina S. Varshavsky M. Kitsberg D. Dvir-Szternfeld R. . (2020). Disease-associated astrocytes in Alzheimer’s disease and aging. Nat. Neurosci. 23, 701–706. doi: 10.1038/s41593-020-0624-8, 32341542 PMC9262034

[ref25] Hao Y. Hao S. Andersen-Nissen E. Mauck W. M. 3rd Zheng S. Butler A. . (2021). Integrated analysis of multimodal single-cell data. Cell 184, 3573–3587.e29. doi: 10.1016/j.cell.2021.04.048, 34062119 PMC8238499

[ref26] Hendrie H. C. Murrell J. Baiyewu O. Lane K. A. Purnell C. Ogunniyi A. . (2014). APOE ε4 and the risk for Alzheimer disease and cognitive decline in African Americans and Yoruba. Int. Psychogeriatr. 26, 977–985. doi: 10.1017/S1041610214000167, 24565289 PMC4012422

[ref27] Hernandez-Quiles M. Broekema M. F. Kalkhoven E. (2021). PPARgamma in metabolism, immunity, and Cancer: unified and diverse mechanisms of action. Front. Endocrinol. (Lausanne) 12:624112. doi: 10.3389/fendo.2021.624112, 33716977 PMC7953066

[ref28] Hohman T. J. Cooke-Bailey J. N. Reitz C. Jun G. Naj A. Beecham G. W. . (2016). Global and local ancestry in African-Americans: implications for Alzheimer’s disease risk. Alzheimers Dement. 12, 233–243. doi: 10.1016/j.jalz.2015.02.012 PMC4681680 26092349

[ref29] Husain M. A. Laurent B. Plourde M. (2021). APOE and Alzheimer's disease: from lipid transport to physiopathology and therapeutics. Front. Neurosci. 15:630502. doi: 10.3389/fnins.2021.630502, 33679311 PMC7925634

[ref30] Huuki-Myers L. A. Spangler A. Eagles N. J. Montgomery K. D. Kwon S. H. Guo B. . (2024). A data-driven single-cell and spatial transcriptomic map of the human prefrontal cortex. Science 384:eadh1938. doi: 10.1126/science.adh1938 PMC11398705 38781370

[ref31] Iqbal K. Grundke-Iqbal I. (2010). Alzheimer's disease, a multifactorial disorder seeking multitherapies. Alzheimers Dement. 6, 420–424. doi: 10.1016/j.jalz.2010.04.006, 20813343 PMC2946155

[ref32] Jiang W. I. Cao Y. Xue Y. Ji Y. Winer B. Y. Chandra R. . (2025). Suppressing APOE4-induced neural pathologies by targeting the VHL-HIF axis. Proc. Natl. Acad. Sci. USA 122:e2417515122. doi: 10.1073/pnas.2417515122 PMC11804744 39874294

[ref33] Joshi S. S. Thieschafer J. Li L. (2025). APOE4 astrocytes exhibit increased susceptibility to aβ, tau, and cytokine-induced mitochondrial dysfunction. Alzheimers Dement. 21:e104761. doi: 10.1002/alz70855_104761

[ref34] Kazmi S. Z. H. Wierenga C. J. (2025). Deep cortical layers are more vulnerable in Alzheimer's disease. Neuron 113, 2218–2220. doi: 10.1016/j.neuron.2025.06.022, 40706559

[ref35] Kim B. Kim D. Schulmann A. Patel Y. Caban-Rivera C. Kim P. . (2023). Cellular diversity in human Subgenual anterior cingulate and dorsolateral prefrontal cortex by single-nucleus RNA-sequencing. J. Neurosci. 43, 3582–3597. doi: 10.1523/JNEUROSCI.0830-22.2023, 37037607 PMC10184745

[ref36] Kumar A. Fontana I. C. Nordberg A. (2023). Reactive astrogliosis: a friend or foe in the pathogenesis of Alzheimer's disease. J. Neurochem. 164, 309–324. doi: 10.1111/jnc.15565, 34931315

[ref37] Lana D. Ugolini F. Iovino L. Attorre S. Giovannini M. G. (2024). Astrocytes phenomics as new druggable targets in healthy aging and Alzheimer's disease progression. Front. Cell. Neurosci. 18:1512985. doi: 10.3389/fncel.2024.1512985, 39835288 PMC11743640

[ref38] LeBleu V. S. O'Connell J. T. Gonzalez Herrera K. N. Wikman H. Pantel K. Haigis M. C. . (2014). PGC-1α mediates mitochondrial biogenesis and oxidative phosphorylation in cancer cells to promote metastasis. Nat. Cell Biol. 16, 992–1003. doi: 10.1038/ncb3039 PMC4369153 25241037

[ref39] Li Z. Luo L. Yu W. Li P. Ou D. Liu J. . (2022). PPARγ phase separates with RXRα at PPREs to regulate target gene expression. Cell Discov. 8:37. doi: 10.1038/s41421-022-00388-0, 35473936 PMC9043196

[ref40] Liu C. C. Liu C. C. Kanekiyo T. Xu H. Bu G. (2013). Apolipoprotein E and Alzheimer disease: risk, mechanisms and therapy. Nat. Rev. Neurol. 9, 106–118. doi: 10.1038/nrneurol.2012.263, 23296339 PMC3726719

[ref41] Luo W. Friedman M. S. Shedden K. Hankenson K. D. Woolf P. J. (2009). GAGE: generally applicable gene set enrichment for pathway analysis. BMC Bioinformatics. 10:161. doi: 10.1186/1471-2105-10-161, 19473525 PMC2696452

[ref42] Luo C. Liu H. Xie F. Armand E. J. Siletti K. Bakken T. E. . (2022). Single nucleus multi-omics identifies human cortical cell regulatory genome diversity. Cell Genom. 2:100107. doi: 10.1016/j.xgen.2022.100107, 35419551 PMC9004682

[ref43] Lysaker C. R. Johnson C. N. Csikos V. Franczak E. Benson M. Gilmore C. A. Proteomic and metabolic profiling reveals APOE4-dependent shifts in whole brain, neuronal, and astrocytic mitochondrial function and glycolysis. bioRxiv [Preprint]. (2025). doi:doi: 10.1101/2025.06.15.659811.

[ref44] Mahley R. W. (2023). Apolipoprotein E4 targets mitochondria and the mitochondria-associated membrane complex in neuropathology, including Alzheimer's disease. Curr Opin Neurobiol 79:102684. doi: 10.1016/j.conb.2023.102684 36753858

[ref45] Mahley R. W. Huang Y. (2012). Apolipoprotein e sets the stage: response to injury triggers neuropathology. Neuron 76, 871–885. doi: 10.1016/j.neuron.2012.11.020, 23217737 PMC4891195

[ref46] Mandrekar-Colucci S. Karlo J. C. Landreth G. E. (2012). Mechanisms underlying the rapid peroxisome proliferator-activated receptor-γ-mediated amyloid clearance and reversal of cognitive deficits in a murine model of Alzheimer's disease. J. Neurosci. 32, 10117–10128. doi: 10.1523/JNEUROSCI.5268-11.201 PMC3433721 22836247

[ref47] Marchiano F. Haering M. Habermann B. H. (2022). The mitoXplorer 2.0 update: integrating and interpreting mitochondrial expression dynamics within a cellular context. Nucleic Acids Res. 50, W490–W499. doi: 10.1093/nar/gkac306, 35524562 PMC9252804

[ref48] Mathys H. Boix C. A. Akay L. A. Xia Z. Davila-Velderrain J. Ng A. P. . (2024). Single-cell multiregion dissection of Alzheimer's disease. Nature 632, 858–868. doi: 10.1038/s41586-024-07606-7 PMC11338834 39048816

[ref49] McKenna A. Hanna M. Banks E. Sivachenko A. Cibulskis K. Kernytsky A. . (2010). The genome analysis toolkit: a MapReduce framework for analyzing next-generation DNA sequencing data. Genome Res. 20, 1297–1303. doi: 10.1101/gr.107524.110, 20644199 PMC2928508

[ref50] Murphy K. B. Hu D. Wolfs L. Rohde S. K. Fauró G. L. Geric I. . (2025). The APOE isoforms differentially shape the transcriptomic and epigenomic landscapes of human microglia xenografted into a mouse model of Alzheimer's disease. Nat. Commun. 16:4883. doi: 10.1038/s41467-025-60099-4, 40419479 PMC12106835

[ref51] Perkins M. Wolf A. B. Chavira B. Shonebarger D. Meckel J. P. Leung L. . (2016). Altered energy metabolism pathways in the posterior cingulate in Young adult apolipoprotein E ɛ4 carriers. J. Alzheimer's Dis 53, 95–106. doi: 10.3233/JAD-151205, 27128370 PMC4942726

[ref52] Qi G. Mi Y. Shi X. Gu H. Brinton R. D. Yin F. (2021). APOE4 impairs neuron-astrocyte coupling of fatty acid metabolism. Cell Rep. 34:108572. doi: 10.1016/j.celrep.2020.108572, 33406436 PMC7837265

[ref53] Rajabli F. Feliciano B. E. Celis K. Hamilton-Nelson K. L. Whitehead P. L. Adams L. D. . (2018). Ancestral origin of APOE ε4 Alzheimer disease risk in Puerto Rican and African American populations. PLoS Genet. 14:e1007791. doi: 10.1371/journal.pgen.1007791, 30517106 PMC6281216

[ref54] Reiman E. M. Caselli R. J. Yun L. S. Chen K. Bandy D. Minoshima S. . (1996). Preclinical evidence of Alzheimer's disease in persons homozygous for the E4 allele for apolipoprotein E. N. Engl. J. Med. 334, 752–758. doi: 10.1056/nejm199603213341202 8592548

[ref55] Sadick J. S. O'Dea M. R. Hasel P. Dykstra T. Faustin A. Liddelow S. A. (2022). Astrocytes and oligodendrocytes undergo subtype-specific transcriptional changes in Alzheimer's disease. Neuron 110, 1788–1805.e10. doi: 10.1016/j.neuron.2022.03.008, 35381189 PMC9167747

[ref56] Sahota A. Yang M. Gao S. Hui S. L. Baiyewu O. Gureje O. . (1997). Apolipoprotein E-associated risk for Alzheimer's disease in the African-American population is genotype dependent. Ann. Neurol. 42, 659–661. doi: 10.1002/ana.410420418, 9382479

[ref57] Schmukler E. Solomon S. Simonovitch S. Goldshmit Y. Wolfson E. Michaelson D. M. . (2020). Altered mitochondrial dynamics and function in APOE4-expressing astrocytes. Cell Death Dis. 11:578. doi: 10.1038/s41419-020-02776-4, 32709881 PMC7382473

[ref58] Serrano-Pozo A. Das S. Hyman B. T. (2024). Astrocyte transcriptomic changes along the spatiotemporal progression of Alzheimer's disease. Nat. Neurosci. 27, 2384–2400. doi: 10.1038/s41593-024-01791-4, 39528672 PMC11614739

[ref59] Shannon P. Markiel A. Ozier O. Baliga N. S. Wang J. T. Ramage D. . (2003). Cytoscape: a software environment for integrated models of biomolecular interaction networks. Genome Res. 13, 2498–2504. doi: 10.1101/gr.1239303, 14597658 PMC403769

[ref60] Shen E. H. Overly C. C. Jones A. R. (2012). The Allen human brain atlas: comprehensive gene expression mapping of the human brain. Trends Neurosci. 35, 711–714. doi: 10.1016/j.tins.2012.09.005 23041053

[ref61] Sienski G. Narayan P. Bonner J. M. Kory N. Boland S. Arczewska A. A. . (2021). APOE4 disrupts intracellular lipid homeostasis in human iPSC-derived glia. Sci. Transl. Med. 13:eaaz4564. doi: 10.1126/scitranslmed.aaz4564, 33658354 PMC8218593

[ref62] Skerrett R. Pellegrino M. P. Casali B. T. Taraboanta L. Landreth G. E. (2015). Combined liver X receptor/peroxisome proliferator-activated receptor γ agonist treatment reduces amyloid β levels and improves behavior in amyloid precursor protein/Presenilin 1 mice. J. Biol. Chem. 290, 21591–21602. doi: 10.1074/jbc.M115.652008, 26163517 PMC4571883

[ref63] Szklarczyk D. Morris J. H. Cook H. Kuhn M. Wyder S. Simonovic M. . (2017). The STRING database in 2017: quality-controlled protein-protein association networks, made broadly accessible. Nucleic Acids Res. 45, D362–D368. doi: 10.1093/nar/gkw937, 27924014 PMC5210637

[ref64] Tambini M. D. Pera M. Kanter E. Yang H. Guardia-Laguarta C. Holtzman D. . (2016). APOE4 upregulates the activity of mitochondria-associated ER membranes. EMBO Rep. 17, 27–36. doi: 10.15252/embr.201540614, 26564908 PMC4718413

[ref65] Tang M. X. Maestre G. Tsai W. Y. Liu X. H. Feng L. Chung W. Y. . (1996). Relative risk of Alzheimer disease and age-at-onset distributions, based on APOE genotypes among elderly African Americans, Caucasians, and Hispanics in New York City. Am. J. Hum. Genet. 58, 574–584. PMC1914582 8644717

[ref66] Theendakara V. Peters-Libeu C. A. Spilman P. Poksay K. S. Bredesen D. E. Rao R. V. (2016). Direct regulation of gene expression by Alzheimer's disease risk factor apolipoprotein E4. Proc. Natl. Acad. Sci. USA 113, 13744–13749. doi: 10.1523/JNEUROSCI.3562-15.2016

[ref67] Trapnell C. Cacchiarelli D. Grimsby J. Pokharel P. Li S. Morse M. . (2014). The dynamics and regulators of cell fate decisions are revealed by pseudotemporal ordering of single cells. Nat. Biotechnol. 32, 381–386. doi: 10.1038/nbt.2859, 24658644 PMC4122333

[ref68] Ulgen E. Ozisik O. Sezerman O. U. (2019). pathfindR: an R package for comprehensive identification of enriched pathways in omics data through active subnetworks. Front. Genet. 10:858. doi: 10.3389/fgene.2019.00858, 31608109 PMC6773876

[ref69] Williams H. C. Farmer B. C. Piron M. A. Walsh A. E. Bruntz R. C. Gentry M. S. . (2020). APOE alters glucose flux through central carbon pathways in astrocytes. Neurobiol. Dis. 136:104742. doi: 10.1016/j.nbd.2020.104742, 31931141 PMC7044721

[ref70] Wu G. Haw R. (2017). Functional interaction network construction and analysis for disease discovery. Methods Mol. Biol. 1558, 235–253. doi: 10.1007/978-1-4939-6783-4_11 28150241

[ref71] Xia J. Gill E. E. Hancock R. E. (2015). NetworkAnalyst for statistical, visual and network-based meta-analysis of gene expression data. Nat. Protoc. 10, 823–844. doi: 10.1038/nprot.2015.052, 25950236

[ref72] Xiong X. Y. Tang Y. Yang Q. W. (2022). Metabolic changes favor the activity and heterogeneity of reactive astrocytes. Trends Endocrinol. Metab. 33, 390–400. doi: 10.1016/j.tem.2022.03.001, 35396164

[ref73] Yilmaz E Chen KW Cakir EÖ Bhattarai P Liu M Ciener BL Single-nucleus multiomics in brains from Hispanic individuals reveal APOE-ε4 -driven disruption of focal adhesion signaling in the presence of cerebrovascular pathology. medRxiv [Preprint]. (2025)

